# Real-world efficacy of brentuximab vedotin plus bendamustine as a bridge to autologous hematopoietic stem cell transplantation in primary refractory or relapsed classical Hodgkin lymphoma

**DOI:** 10.1007/s00277-020-04204-1

**Published:** 2020-08-03

**Authors:** László Imre Pinczés, Roxána Szabó, Árpád Illés, Dóra Földeák, Klára Piukovics, Árpád Szomor, László Gopcsa, Zsófia Miltényi

**Affiliations:** 1 Division of Hematology, Department of Internal Medicine, Faculty of Medicine, University of Debrecen, Nagyerdei krt. 98, Debrecen, 4032 Hungary; 2 Doctoral School of Clinical Medicine, University of Debrecen, Debrecen, Hungary; 3 Division of Hematology, 2nd Department of Internal Medicine, Faculty of Medicine, University of Szeged, Szeged, Hungary; 4 Division of Hematology, 1st Department of Internal Medicine, Faculty of Medicine, University of Pécs, Pecs, Hungary; 5 Department of Hematology and Stem Cell Transplantation, Central Hospital of Southern Pest National Institute of Hematology and Infectious Diseases, Budapest, Hungary

**Keywords:** Hodgkin lymphoma, Autologous hematopoietic stem cell transplantation, Brentuximab vedotin, Bendamustine, Survival

## Abstract

Up to 30% of patients with classical Hodgkin lymphoma (cHL) are not responsive to frontline therapy or relapse after primary treatment. In these cases, autologous hematopoietic stem cell transplantation (AHSCT) is the standard of care. The combination of brentuximab vedotin and bendamustine (BV + B) is an effective salvage regimen in this challenging subpopulation. This nationwide multicenter study investigated the real-world efficacy and safety of the BV + B regimen as a bridge to AHSCT in patients with primary refractory or relapsed cHL. A total of 41 cHL patients underwent AHSCT after receiving at least 1 cycle of BV + B (with brentuximab vedotin given at 1.8 mg/kg on day 1 and bendamustine at 90 mg/m^2^ on days 1–2 every 4 weeks). After a median of 3 (1–6) cycles of BV + B, the objective response rate was 78%, with 29 (70.7%) patients achieving complete remission. Twelve (29.3%) patients relapsed after AHSCT, 2 (4.9%) of them died, while 2 (4.9%) patients are lost to follow-up. After a median of 17 months of follow-up, the estimated 2-year overall- and progression-free survival after AHSCT was 93 and 62%, respectively. Features of advanced disease at recurrence (*p* = 0.038) and the presence of stage IV cHL at relapse (*p* = 0.024) are strong predictor markers of unfavorable outcomes. Twenty-four (58.5%) patients experienced adverse events of any grade, while no grade IV toxicities were reported. BV + B is an effective salvage option with a manageable toxicity profile in cHL. The real-world safety and efficacy of this combination are similar to the observations made on the study population.

## Introduction

With the new risk- and response-adapted treatment modalities, classical Hodgkin lymphoma (cHL) became a highly curable hematologic malignancy, with 80–90% of patients achieving long-term remission after standard first-line therapy [1, 2]. However, 20–30% of cHL patients have primary refractory disease or will experience recurrence. In these patients, an autologous hematopoietic stem cell transplantation (AHSCT) is the standard of care, despite the 50% relapse rate after transplantation in cHL [3]. Several prognostic factors associated with an increased risk of relapse following AHSCT include primary refractory cHL, stage IV disease at relapse, extranodal involvement, presence of B symptoms, and less than a complete remission (CR) to salvage therapy before AHSCT [4]. Achievement of CR by positron emission tomography/computed tomography (PET/CT) before AHSCT is a strong predictor for a favorable outcome [5–9].

Complete remission rates before AHSCT with conventional salvage chemotherapy regimens, such as DHAP (cisplatin, cytarabine, and dexamethasone), ESHAP (etoposide, methylprednisolone, cytarabine, and cisplatin), IGEV (ifosfamide, gemcitabine, etoposide, and vinblastine), BeGEV (bendamustine, gemcitabine, and vinorelbine), and ICE (ifosfamide, carboplatin, and etoposide) vary from 17 to 76% [5, 6, 9–11]. In recent years, novel therapies (brentuximab vedotin, anti-programmed cell death-1 (PD-1) inhibitors) became available to help improve transplant outcomes and also survival rate of patients relapsing after AHSCT.

One of the novel combination therapies is brentuximab vedotin (BV) plus bendamustine (BV + B). BV is an antibody-drug conjugate, which consists of an anti-CD30 chimeric monoclonal antibody and the microtubule-disrupting agent, monomethyl auristatin E. Bendamustine is a bifunctional molecule containing the alkylating agent nitrogen mustard and the purine analog fludarabine, causing intra- and inter-strand cross-links between DNA bases resulting in cell death. In heavily pretreated cHL patients, BV used as monotherapy resulted in CR rates and overall response rates (ORR) of 27–35 and 72–75%, respectively [12–14]. CR and OR rates associated with single-agent bendamustine therapy were 33 and 53% [15]. The combination of these two agents is outstanding in a practical way. The increased proportion of patients achieving CR (43–74%), reduced toxicity burden compared with standard platinum-based salvage protocols, nonoverlapping toxicities of the combined agents, and the opportunity to treat patients in the outpatient setting highlight BV + B regimen, compared with other BV-based therapies [10, 16–18].

According to the European Medicines Agency (EMA), brentuximab vedotin is indicated for the treatment of adult cHL patients with relapsed or refractory cHL after AHSCT or following at least two prior therapies when AHSCT or multiagent chemotherapy is not a treatment option or for cHL patients at increased risk of relapse or progression following AHSCT [19]. Therefore, in everyday practice, brentuximab vedotin can be used as a second salvage therapy for relapsed or refractory cHL patients as a sole agent, or even in combination.

The purpose of this study was to evaluate the safety and efficacy of the BV + B combination therapy as a bridge to transplantation in relapsed or refractory cHL patients who previously received two or more multiagent chemotherapy regimens.

## Methods

### Study design and participants

**Fig. 1:**
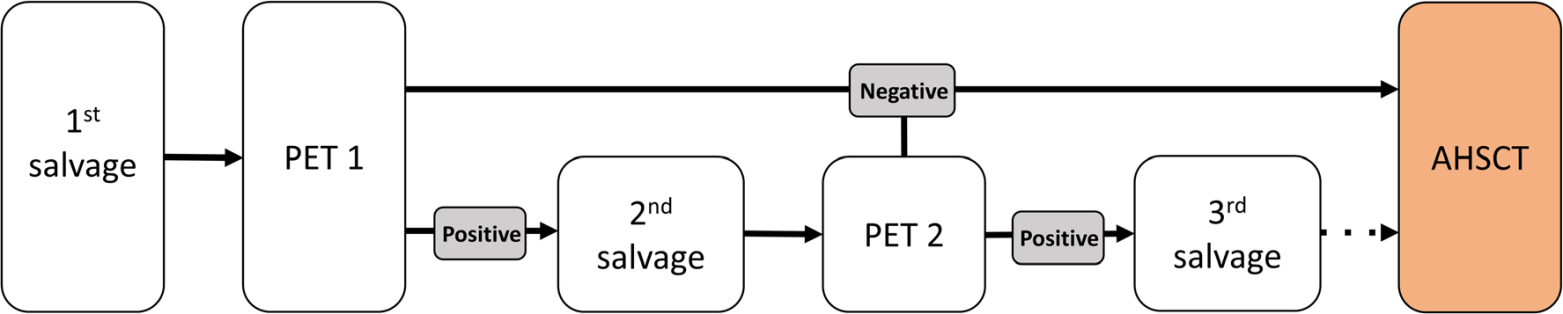
Practice guideline of the Hungarian Society of Hematology and Transfusion for the treatment of primary refractory or relapsed classical Hodgkin lymphoma. Abbreviations: PET, positron emission tomography; AHSCT, autologous hematopoietic stem cell transplantation

We retrospectively analyzed the demographic data and clinical features of cHL patients receiving BV + B salvage therapy before AHSCT, between January 01, 2016, and December 31, 2018, treated at the four national transplant centers: the University of Debrecen (Debrecen, Hungary), the University of Pecs (Pecs, Hungary), the University of Szeged (Szeged, Hungary), and the Central Hospital of Southern Pest National Institute of Hematology and Infectious Diseases (Budapest, Hungary). Patients were treated according to the evidence- and consensus-based practice guidelines of the Hungarian Society of Hematology and Transfusion (Fig. 1) [20].

Eligible patients were aged 18 years or older and had a histologically confirmed diagnosis of classical cHL. Patients must have had relapsed or refractory disease following standard first-line polychemotherapy. We included patients who received at least one cycle of BV + B regimen in guideline-based dose. No exclusion criteria were determined regarding marrow and other organ function, Eastern Cooperative Oncology Group (ECOG) performance status, or a total number of previous therapies received.

All patients provided written informed consent during enrollment. Local research ethics committees of all participating centers approved the study, which was done according to the Declaration of Helsinki.

### Procedures and assessment

Relapsed or refractory cHL patients received at least 2 cycles of a standard salvage chemotherapy regimen before the administration of BV + B combination therapy. Patients received 1 to 6 cycles of BV + B with a dose of 1.8 mg/kg brentuximab vedotin intravenously on day 1 and 90 mg/m^2^ of bendamustine intravenously on each of days 1 and 2 of a 21-day cycle. AHSCTs were performed with BEAM (carmustine, etoposide, cytarabine, and melphalan) conditioning regimen.

Response to the salvage therapies was assessed using the 2016 Refinement of the Lugano Classification Lymphoma Response Criteria [21]. Failure after at least one standard salvage chemotherapy regimen made relapsed or refractory cHL patients eligible for BV + B therapy. A dedicated PET/CT scan was performed after cycle 2 of BV + B combination therapy, and later as it was deemed necessary. PET-negative patients (Deauville score 1–3) underwent AHSCT at any time after cycle 2, while PET-positive (Deauville score 4–5) patients were administered further antitumor therapy.

Stem cell mobilization and collection and also the administration of standard supporting treatment were performed according to institutional guidelines. Adverse events (AE) were monitored at every visit throughout treatment and follow-up.

### Outcomes

Our analysis focuses on the CR and OR rates of relapsed or refractory cHL patients treated with BV + B combination therapy before AHSCT. Treatment response rates were also evaluated regarding PET/CT status according to the Lugano Classification. The overall survival (OS) was calculated from the day of AHSCT to the last follow-up visit or death. Progression-free survival (PFS) was defined as the time from AHSCT to disease progression, to relapse, or to death. Statistical analysis was performed via Fisher’s exact test, and survival data were calculated using the Kaplan-Meier method, with the SPSS 25.0 software.

## Results

### Patient characteristics and treatment

During the 3-year observational period, 41 cHL patients with relapsed or refractory cHL underwent AHSCT after receiving BV + B salvage therapy (Table 1). The majority (61%) of patients had nodular sclerosing cHL, and a marked male predominance was present. Thirty-two (78%) patients had an advanced-stage disease at initial diagnosis. All patients had received ABVD as frontline therapy, in accordance with the national guidelines. Twenty-eight (68.3%) patients had primary refractory disease. At relapse, 23 (56%) patients had stage III–IV disease, while 8 (19.5%) of them had extranodal involvement.

**Table 1:** Patient characteristics

	Patients	%
Men	25	61%
Women	16	39%
Histological subtypes		
MC	6	14.6%
NS	25	61%
LR	5	12.2%
LD	1	2.4%
ND	4	9.8%
Stage at the diagnosis		
II	8	19.5%
III	11	26.8%
IV	21	51.2%
Refractory	28	68.3%
Relapse ≤ 12 months	9	22%
Relapse > 12 months	4	9.7%
Stage at relapse		
I	1	2.4%
II	16	39%
III	9	22%
IV	14	34.1%
Extranodal involvement	8	19.50%
B symptoms	15	36.60%
Number of salvage therapies		
2	24	58.5%
≥ 3	17	41.5%
PET − (before AHSCT)	29	70.7%
PET + (before AHSCT)	12	22%
Relapse after AHSCT	12	29.3%
Alive	37	90.2%
Dead	2	4.9%
Lost to follow-up	2	4.9%

*MC* mixed cellularity, *NS* nodular sclerosing, *LR* lymphocyte rich, *LD* lymphocyte depleted, *ND* not defined, *PET* positron emission tomography, *AHSCT* autologous hematopoietic stem cell transplantation

The median number of prior salvage therapies preceding BV + B was 3 (range 1–6). Twenty (48.8%) patients received DHAP, while 4 (9.8%) patients received ifosfamide-based first salvage regimen. Seventeen (41.5%) patients received two or more salvage therapies before BV + B, including DHAP, ESHAP, IGEV, and PD-1 inhibitor. Patients received a median of 3 (range 1–6) cycles of BV + B. The last salvage regimen before AHSCT was BV + B.

### Treatment response and long-term follow-up

Of the 41 evaluable patients, 29 (70.7%) achieved CR with BV + B therapy before AHSCT. The ORR was 92.6% overall, with 9 (21.9%) patients having partial remission (PR). Twenty-nine (70.8%) patients were PET-negative, and 12 (29.2%) patients were PET-positive before AHSCT. Of the 14 patients with stage IV disease at cHL progression or relapse, the CR and ORR rates were 64.3 and 85.7%, respectively.

**Fig. 2:**
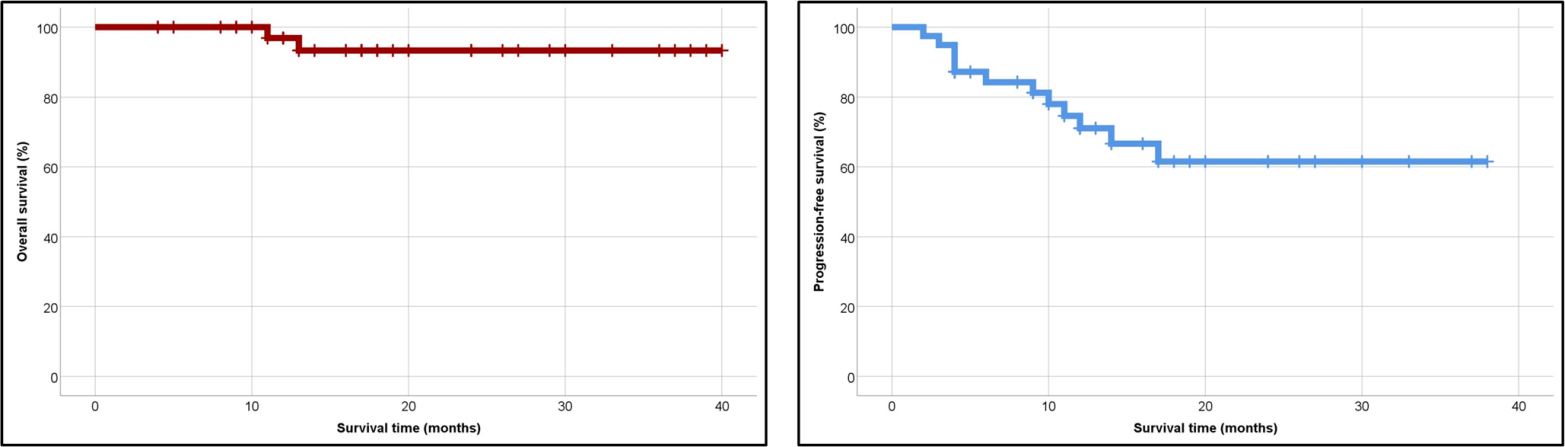
Median 2-year overall- and progression-free survival for all patients. Abbreviation: Tx, transplantation

**Fig. 3:**
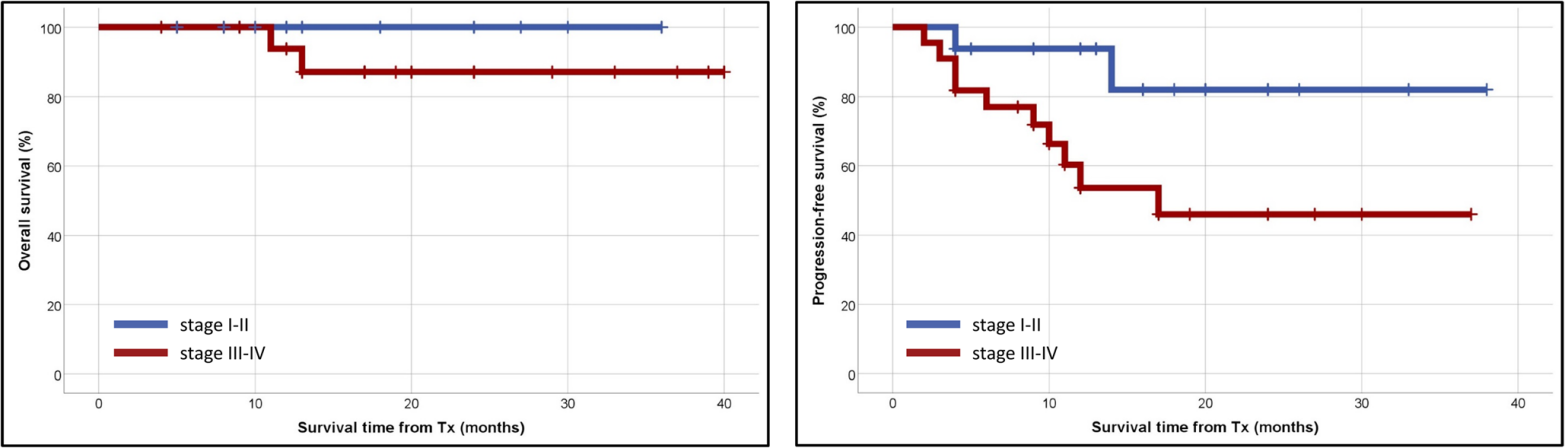
Median 2-year overall- and progression-free survival according to disease stage at recurrence (stage I–II vs. stage III–IV). Abbreviation: Tx, transplantation

**Fig. 4:**
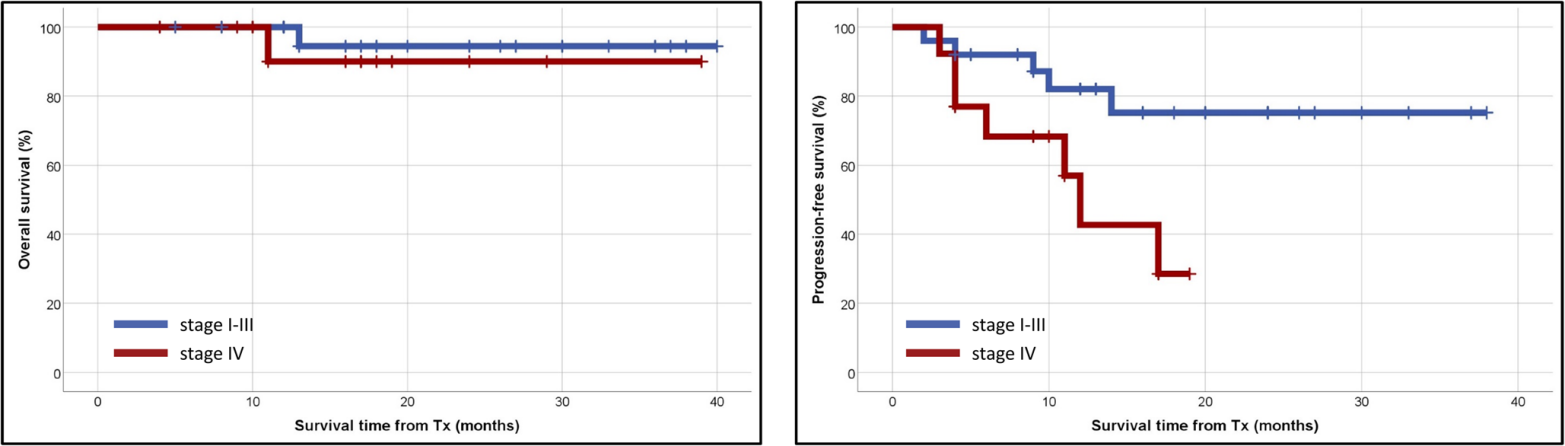
Median 2-year overall- and progression-free survival according to disease stage at recurrence (stage I–III vs. stage IV). Abbreviation: Tx, transplantation

Twelve (29.2%) patients relapsed after AHSCT, including 8 (19.5%) patients who underwent AHSCT with PET-negative cHL. With a median follow-up of 17 (range 2–40) months, 37 patients are alive, two patients died, and two have been lost to follow-up. One patient died of disease progression and one of septic shock. None of the deaths were considered treatment-related. The median 2-year OS and PFS were 93 and 62%, respectively (Fig. 2). Compared with patients with stage I–II cHL at relapse, patients with advanced disease features at recurrence had an inferior outcome (*p* = 0.038) (Fig. 3). Also, the presence of stage IV cHL at relapse is a strong predictor marker of unfavorable outcome (*p* = 0.024) (Fig. 4). It is noteworthy that the survival curves reach a plateau before 18 months of follow-up. No association was found between outcome and any of the following features: age, sex, B symptoms, or histological subtype. Also, patients who achieved PET negativity before AHSCT had no survival benefit compared with the PET-positive group.

Thirty-seven patients who underwent AHSCT were at increased risk of relapse or progression, based on the EMA indication criteria. However, only 15 patients received additional, posttransplant BV monotherapy. Eleven of the 12 PET-positive patients were candidates for single-agent BV after AHSCT, but only two of them were treated. Also, 13 of the 26 available patients received BV maintenance in the PET-negative group.

### Adverse events

A total of 24 (58.5%) patients experienced treatment-related adverse events (AE) of any grade (Table 2). The most common toxicities were neutropenia (17%), peripheral neuropathy (12.2%), and infusion-related reactions (IRR) with fever, chills, flushing, or pruritus (12.2%). No cases of anaphylaxis were recorded. There were no grade 4 toxicities, and only a total of 3 (7.3%) patients experienced grade 3 toxicities. Serious AEs were neutropenia in 2 (4.8%) patients and peripheral neuropathy in 1 (2.4%) patient. One patient discontinued bendamustine due to severe, treatment-related neutropenia. Patients did not receive prophylactic corticosteroids or growth factor support routinely.

**Table 2:** Summary of treatment-related adverse events

Adverse events	Patients	%
Neutropenia	7	17%
Peripheral neuropathy	5	12.2%
Infusion-related reaction	5	12.2%
Bronchitis, pneumonia	2	4.9%
GI	2	4.9%
Rash	1	2.4%
CMV infection	1	2.4%
Herpes zoster infection	1	2.4%

*CMV* cytomegalovirus, *GI* gastrointestinal

## Discussion

To date, three prior phase 1–2 studies and a retrospective analysis evaluated the combination of BV and bendamustine in relapsed or refractory cHL patients. BV + B regimen showed marked activity in a heavily pretreated population of patients. LaCasce et al. reported on 55 cHL patients who relapsed after first-line chemotherapy and were treated with BV + B within a multicenter, phase 2 trial [16]. The overall response and CR rates were 92.5 and 73.6%, respectively. Those 40 patients, who proceeded to AHSCT, had improved OR (95%) and CR (85%) rates, with a 2-year OS of 94.9% and a 2-year PFS of 69.8%. More than half (56.4%) of this patient population experienced grade 3–4 AEs, with lymphopenia, rash, and hypotension occurring most frequently. The incidence of an infusion-related reaction, defined as fever, chills, dyspnea, flushing, nausea, pruritus, hypotension, or the combination of these, was 60%, which is more than single-agent brentuximab vedotin or bendamustine caused alone (12–15%) [22, 23]. Peripheral neuropathy occurred in 54.4% of the evaluable patients. O’Connor et al. treated 37 patients with an ORR of 78% in a phase 2 study population [17]. Forty-three percent of these patients achieved a complete response, while the 2-year OS and PFS were 80 and 62%, respectively. The most common grade 3–4 AEs were neutropenia (35%) and lung infection (14%). Broccoli et al. also observed high remission rates (ORR 80%, CR 75%) and promising 3-year OS and PFS (88.1 and 67.3%, respectively) with BV + B in 40 cHL patients, who inadequately responded to standard induction [18]. Martineau et al. administered BV + B combination to 80 heavily pretreated, relapsed, or refractory cHL patients. They reported a CR in 49 (65%) of 76 patients evaluable for efficacy, with an estimated 2-year OS and PFS of 88.5 and 64%, respectively. Patients eligible to AHSCT had an improved posttransplant CR rate (81%), compared with patients in the group without AHSCT (49%). The most frequent (> 30%) toxicities were hematological and infectious [24].

Our results are similar to these data in terms of response rates, estimated survival, and toxicities (Table 3). Seemingly, cHL patients who do not respond to one or more traditional chemotherapy regimens could be effectively treated with BV + B salvage therapy and consolidated by AHSCT. However, it is important to note that, according to the national regulations on BV indication, we used BV + B combination as second salvage therapy. Notably, the majority of post-AHSCT relapses occur in the first year after transplantation. Around 18 months, the Kaplan-Meier PFS survival curve for all patients began to plateau and extended to 38 months for the longest survival follow-up.

**Table 3:** Observations with brentuximab vedotin plus bendamustine combination in primary refractory or relapsed classical Hodgkin lymphoma

Authors	Reference no.	No. of patients	ORR (%)	CR (%)	2-year PFS (%)
LaCasce et al.	16	55	92.5	73.6	62.6
O’Connor et al.	17	37	78.0	43.0	62.0
Broccoli et al.	18	40	80.0	75.0	67.3
Martineau et al.	24	80	n/a	65.0	64.0
Pinczés et al.	–	41	92.7	70.8	62.0

*et al.* et alia, *no.* number, *ORR* overall response rate, *CR* complete remission, *PFS* progression-free survival, *n/a* not available

Achievement of a negative PET/CT scan before AHSCT had no impact on PFS, which may be due to the low number of patients included in the analysis. Also, because of the expanse in therapeutic options, all cHL patients are transplanted in the deepest achievable metabolic remission. Positive PET/CT results predominantly represent localized or non-widespread disease activity. In these cases, a high-dose conditioning regimen before AHSCT or posttransplant consolidation therapy can also be curative.

The impact of BV maintenance therapy for patients with high risk for relapse after AHSCT is challenging to assess. According to the AETHERA trial, high-risk patients are the ones with primary refractory HL, relapsed HL with an initial remission duration of less than 12 months, or extranodal involvement at the start of pre-transplantation salvage chemotherapy. While most patients were candidates according to this criteria, only 38% of them received BV consolidation, and there was no difference in either PFS or OS compared with low-risk cHL patients. The lack of proven survival benefit may also be the result of BV administration before AHSCT, as it provides better survival rates used as salvage therapy than conventional chemotherapy regimens. However, based on the results of the AETHERA study, consolidation treatment with BV is strongly recommended, as it significantly improved PFS compared with the placebo arm (5-year PFS of 59% vs. 41%, respectively) [25]. The low proportion of BV maintenance among the high-risk patients of this analysis is due to drug availability issues.

In relapsed or refractory cHL patients, several standard salvage chemotherapy regimens (ICE, DHAP, ESHAP) and checkpoint inhibitors (nivolumab, pembrolizumab) were complemented with BV in early-phase studies, resulting in OR and CR rates ranging from 68 to 100% and 34–100%, respectively [11, 26]. Also, bendamustine-based BeGEV regimen reached OR and CR rates comparable with those achieved with BV + B combination and is considered a feasible candidate for first salvage in primary refractory or relapsed cHL. However, the possibility arises that the effectiveness of a subsequent treatment with BV + B would be impaired in BeGEV-resistant patients. Along with the achievement of a durable disease control and the favorable safety profile, the main advantage of the BV + B regimen, compared with the combinations mentioned above, is the benefit of the administration in the outpatient setting, resulting in the improvement of quality of life.

However, the results of this study suggest that patients with advanced stages, particularly with stage IV cHL at relapse, have inferior outcomes compared with relapsed or refractory cHL patients with early-stage cHL at recurrence. These patients might be appropriate candidates for BV combined with traditional chemotherapy regimens (e.g., augmented ICE) or other novel therapies (e.g., nivolumab) to achieve significantly improved PFS.

Potential limitations of our analysis include the retrospective nature of data collection, limiting the ability to determine cause and effect. Also, due to the relatively low number of patients receiving consolidation, we were not able to assess the impact of BV maintenance therapy. However, we believe that the inclusion of patients from all age groups with no regard to co-morbidities represents real-world experience and can be considered the main strength of the current report.

The treatment paradigm of relapsed and refractory cHL has changed with the availability of BV and checkpoint inhibitors. With the successful introduction of these novel agents into salvage therapy, there will be another shift in treatment, with these agents being incorporated into first-line regimens in the future. Also, the indication of radiation therapy has already been significantly reduced. The use of the BV + B regimen as a bridge to AHSCT in relapsed or refractory cHL patients can be an outstanding example of this process. BV + B is a promising, highly active salvage option with a manageable toxicity profile and a potential for long-term disease control. Complemented by AHSCT, BV + B regimen has the potential to considerably improve the outcome of cHL patients progressing after first-line therapy. The comparison of BV + B with other salvage regimens demands prospective analysis.

## Data Availability

The datasets generated during and/or analyzed during the current study are available from the corresponding author on reasonable request.
